# The Promotion Strategy of Green Construction Materials: A Path Analysis Approach

**DOI:** 10.3390/ma8105354

**Published:** 2015-10-14

**Authors:** Chung-Fah Huang, Jung-Lu Chen

**Affiliations:** Department of Civil Engineering, National Kaohsiung University of Applied Sciences, No.415, Chien-Kung Rd., Kaohsiung City 807, Taiwan; e0955006007@yahoo.com.tw

**Keywords:** green material, dry-mix mortar, carbon emission, path analysis, common method variance

## Abstract

As one of the major materials used in construction, cement can be very resource-consuming and polluting to produce and use. Compared with traditional cement processing methods, dry-mix mortar is more environmentally friendly by reducing waste production or carbon emissions. Despite the continuous development and promotion of green construction materials, only a few of them are accepted or widely used in the market. In addition, the majority of existing research on green construction materials focuses more on their physical or chemical characteristics than on their promotion. Without effective promotion, their benefits cannot be fully appreciated and realized. Therefore, this study is conducted to explore the promotion of dry-mix mortars, one of the green materials. This study uses both qualitative and quantitative methods. First, through a case study, the potential of reducing carbon emission is verified. Then a path analysis is conducted to verify the validity and predictability of the samples based on the technology acceptance model (TAM) in this study. According to the findings of this research, to ensure better promotion results and wider application of dry-mix mortar, it is suggested that more systematic efforts be invested in promoting the usefulness and benefits of dry-mix mortar. The model developed in this study can provide helpful references for future research and promotion of other green materials.

## 1. Introduction

Many countries have dedicated more efforts to reducing anthropogenic CO_2_ emissions since signing of Kyoto Protocol in 1997 [1,2]. However, the problem of global warming is continuously worsening while the construction industry is a major contributor to heavy pollutions and energy consumption [3]. Based on the estimation by the United Nations Environment Programme, the building sector accounts for 30%–40% of global energy use [4]. In addition, the CO_2_ emissions from embodied energy of construction constitute an important share of the total, indicating that building materials have a high importance which is often ignored. Traditional cement is a major source of carbon emissions among the materials used in construction. Besides, transportation and use of cement mortar can lead to heavy air pollutions with flying particles. Therefore, how to improve carbon emissions and environmental pollution problems is an issue of great importance.

A traditional cement mortar is produced by mixing a certain ratio of water, sand and cement according to the experiences of the workers. Such a method requires relative large space of material storage and maneuver and often causes a serious pollution problem of flying particles. In addition, this method often leads to material losses and waste of materials [5]. To reduce the pollutions and inconveniences caused by traditional cement mortar, dry-mix mortar [6] was developed in Western Europe in the 1960s to solve the shortcomings of on-site mixed mortar [7]. Dry-mix mortar is factory-mixed with a fixed ratio of all the powder-form ingredients, such as cement, ground granulated blast-furnace slag, silicon ash, sand and other additives [8]. Different dry-mix mortar manufacturers have different formula and most of the formulas are trade secrets. Users can use it directly by mixing it with a certain ratio of water [9]. This material has the benefits of centralized production, clean processing, and prevention of problems caused by on-site mixing. In addition, dry-mix mortar is also more environmentally friendly than traditional cement mortar and much helpful for emission reduction [10,11].

## 2. Method

Promoting green materials, like dry-mix mortar, is not easy in the traditional construction industry. To prove the potential of dry-mix mortar on carbon-emission reduction and find out the strategy to promote this material, this study employs both qualitative and quantitative methods: a case study and a statistical path analysis. The case study illustrates a typical residential building to calculate and estimate the contribution of using dry-mix mortar to CO_2_ reduction in Taiwan. The statistical framework of this study is based on the theory of technology acceptance model (TAM) proposed by [12]. TAM discusses the use and promotion of a technology from the perspective of users. It has been widely used in many studies, except for those related to the construction industry. TAM mainly explores the influences of external factors on internal factors of users, such as their beliefs, attitudes and intentions toward using a technology, and the influences of these internal factors on their use of the technology. During the past two decades, TAM has been used in many studies and generated many important findings. For example, in the study on the motivation of using computers [13], it was found that perceived ease of use and usefulness of a computer system were both positively and significantly correlated with all the factors related to the use of the computer system (frequency, time length, and amount of tasks) while perceived ease of use had a significant and direct influence on perceived usefulness. The empirical study [14] found that, after users actually used or learned about the use of a new technology, their perceived usefulness of the technology had a direct influence on the level of their use of this technology. There are six most common component dimensions of TAM [12].

- External Variables (EV): external factors that have a potential influence on user’s perceived ease of use and usefulness of a technology, such as their subjective perceptions, or the characteristics and structural organization of the technology or system. With the inclusion of this dimension, TAM has become more mature and complete in explaining and predicting the acceptance of a technology.
- Perceived Usefulness (PU): the degree to which a person believes that using a particular system would enhance his or her job performance.
- Perceived Ease Of Use (PEOU): the degree to which a person believes that using a particular system would be free from effort.
- Attitude toward using (A): the strength of a person’s positive or negative feeling of using an information technology. If his attitude toward a behavior is stronger, he is more likely to conduct the behavior. Therefore, one’s attitude toward a behavior can be used to precisely predict his behaviors.
- Behavioral Intention to use (BI): intentions of the users to use a technology or system. Higher PU and PEOU will result in higher BI, which will then result in higher Actual System Use.
- Actual System Use (ASU), which is subject to the influence of BI. Higher BI will result in higher ASU; and TAM allows researchers to explore the influences of external factors on a user’s beliefs, attitudes, and intention and then predict his use of technologies. External factors such as personal variances, system characteristics, environmental variances, can affect a user’s beliefs by affecting his PU and PEOU of a technology or system. In the past research, there was a continuing controversy over the connections among PU, A, BI and ASU. After the revisions [15,16], BI was excluded from TAM and the exclusion was supported by many research findings. In this research, the dimension of EV covers the past experience (PE) of the users and the quality characteristics (QC) of dry-mix mortar. In addition, the dimensions of PU and PEOU are also used to analyze the subject’s use of dry-mix mortar.

The subjects in this study are people from the construction industry in Taiwan who have experiences of using dry-mix mortar, including people working for construction companies, subcontractors, architect design firms and construction material suppliers. They were invited to answer the questionnaires anonymously in this study. In total, 210 questionnaires were sent and 180 valid samples were returned. To improve the expertise validity of the questionnaire survey, five experts who have over ten-year experiences of using mortars were selected to pretest the questionnaire. After the data collection and further analysis, these experts were interviewed for the discussion in the research implications.

The questionnaire uses a 5-point Likert scale to measure the respondent’s attitudes and perceptions in each dimension. The respondents indicate the level of their agreement of the statement in each question, with one point for “strongly disagree” and five points for “strongly agree”. The questions cover the six dimensions: PE, QC, PU, PEOU, A and ASU. The questionnaire results are then statistically analyzed using SPSS software to conduct reliability analysis, correlation analysis, and path analysis.

## 3. Case Study: CO_2_ Emission Reduction of Dry-Mix Mortar

**Figure 1:**
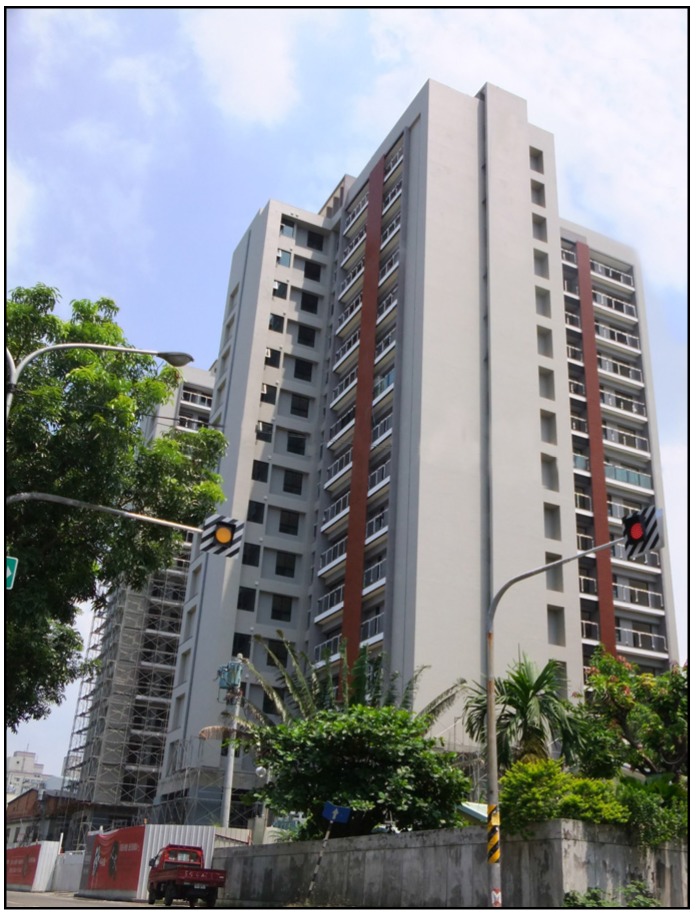
The Residential Building as the Case in this Study.

To illustrate the potential contribution of dry-mix mortar to emission reduction, a typical residential building in Taiwan with two underground floors and 12 aboveground floors with a total floor area of 21,243 m^2^ (see Figure 1) is used as a case in this research. Dry-mix mortar was used in all the plastering of the building. This study assumes ground granulated blast-furnace slag (GGBS) is used to replace the 10% of cement used in the mortar, which is the best ratio for dry-mix mortar production. The amount of carbon emissions of GGBS used in dry-mix mortar is based on the data provided by independent BIS certification agencies commissioned by the representative dry-mix mortar manufacturers.

This case uses dry-mix mortar in the plastering of interior walls (747 tons), exterior walls (214.6 tons) and interior floors (424.8 tons). Based on the international Gabi database [17], the carbon emissions of traditional cement clinkers are 773.7 kg/ton, so carbon emissions of dry-mix mortar is 773.7 kg/ton – (773.7 kg/ton × 10%) + (52.2 kg/ton × 10%) = 701.5 kg/ton.

In this case, the carbon emissions from traditional cement clinker for plastering per one square meter is equal to 50.5 kg/m^2^ (1072.7 tons/21,243 m^2^) while the carbon emissions from dry-mix mortar clinker for plastering per one square meter is 45.8 kg/m^2^ (972.6 tons/21,243 m^2^).

According to the Construction and Planning Agency of Ministry of the Interior, the total floor area of buildings in Taiwan that have received construction licenses in 2011 is 34,148,423 m^2^. The results are shown as follows in Table 1. Based on the floor area of this building and on the total floor area of all the buildings under construction in one year, it can be estimated that using dry-mix mortar in plastering in Taiwan alone can reduce carbon emissions by 160,497.6 tons. This impressive figure of carbon emission reduction is just an estimate, not including the possible reduction from the civil engineering. In Taiwan, civil engineering accounts for 50% of the output value of the whole construction industry. There is still much room and potential for promotion of using dry-mix mortar in civil engineering projects.

**Table 1:** CO_2_ Emission Comparison between Traditional Cement Mortar and Dry-mix Mortar.

Material	CO_2_ Emission	Construction Project of A Typical Building in Taiwan				Total CO_2_ Emission from Total Residential Buildings
		CO_2_ Emission From Interior Wall Plastering	CO_2_ Emission From Exterior Wall Plastering	CO_2_ Emission From Interior Floor Plastering	Total CO_2_ Emission	
**Traditional Cement Mortar**	773.7 *kg/t*	747 *t* × 773.7 *kg/t* = 578.0 *t*	214.6 *t* × 773.7 *kg/t* = 166.0 *t*	424.8 *t* × 773.7 *kg/t* = 328.7 *t*	1072.7 *t*	34,148,423 *m^2^* × 50.5 *kg/m^2^* = 1,724,495.3 *t*
**Dry-mix Mortar**	701.5 *kg/t*	747 *t* × 701.5 *kg/t* = 524.0 *t*	214.6 *t* × 701.5 *kg/t* = 150.6 *t*	424.8 *t* × 701.5 *kg/t* = 298.0 *t*	972.6 *t*	34,148,423 *m^2^* × 45.8 *kg/m^2^* = 1,563,997.7 *t*
**Reduced CO_2_ Emission**	72.2 *kg/t*	54 *t*	15.4 *t*	30.7 *t*	100.1 *t*	160,497.6 *t*

## 4. Statistical Results

### 4.1. Reliability and Correlation Analysis

There are in total 34 items in the questionnaire covering the six dimensions of TAM. All the dimensions have Cronbach’s α values over 0.70, indicating high consistency and stability of the questionnaire results [18,19,20]. The results are shown as follows in Table 2.

**Table 2:** Reliability coefficient of perceptions in each dimension.

Dimension	Cronbach’s α
Past experience (PE)	0.903
Quality characteristics (QC)	0.896
Perceived Usefulness (PU)	0.898
Perceived Ease Of Use (PEOU)	0.896
Attitude toward using (A)	0.892
Actual System Use (ASU)	0.908

This research uses Pearson correlation analysis to test the correlations among the dimensions in its research model and finds the dimensions discussed in this study are positively and significantly correlated with each other [21]. In particular, QC and PU (r = 0.688**), PEOU and PU (r = 0.661**), PEOU and A (r = 0.640**), and A and ASU (r = 0.649**) are highly positively correlated with each other. According to this finding, it can deduced that the dimension of QC is probably helpful in improving the subject’s PE and PEOU of dry-mix mortar and, consequentially, improving their A and ASU regarding dry-mix mortar. Relatively high correlation coefficients may be a result of so-called “common method variance” (CMV), in which the questionnaire respondents are likely to categorize unrelated concepts into the same category, causing unnecessary correlations between two unrelated concepts. To avoid this problem, the Harman’s one-factor test is used in this study to analyze each question in the questionnaire with the number of factors set at one and all the questions unrotated [22]. According to the test results, the explained variance ratio is 40.65%, still less than 50%, indicating there was no obvious problem of CMV in the subject data in this study.

### 4.2. Path Analysis

**Figure 2:**
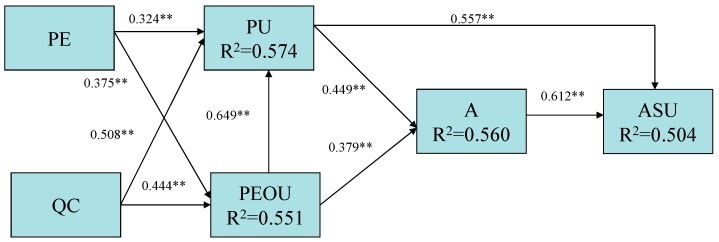
Path analysis of revised technology acceptance model (TAM).

Correlation analysis can only measure the levels of correlation among variables but not the causalities between variables. Thus, path analysis has to be used to find out if there is any causality between two or more variables in the theoretical model [23]. Path analysis can be seen as a single-variable form of structural equation model because it only considers the observed variables without considering any potential variable. The analysis method used to test the theoretical model in this study is composed of a series of regression analyses that allow all the predicted variables to enter the regression model. Therefore, it can be seen as a composition of several multiple regression Equations. The theoretical model in this study is composed of five parts shown as follows. The coefficients, b1 to b9, are standardized versions of linear regression weights representing the effects of independent variables on dependent variable in each equation individually. The first part covers the influence of past experience and quality characteristics on the PU dimension with the PU as the dependent variable and the past experience and quality characteristics as the independent variables. The second part covers the influence of past experience and quality characteristics on the PEOU with the PEOU as the dependent variable and the past experience and quality characteristics as independent variables. The third part covers the influence of PU and PEOU on the attitude with the attitude as the dependent variable and the PU and PEOU as independent variables. The fourth part covers the influence of PU and attitude on the actual system use with the actual system use as the dependent variable and the PU and attitude as the independent variables. The five parts of the model are expressed as the following Equations, and the path diagram of this study is illustrated in Figure 2.

(1) PU = b1PE + b2QC + ε1

(2) PEOU = b3PE + b4QC + ε2

(3) PU = b5PEOU+ ε3

(4) A = b6PU + b7PEOU + ε4

(5) ASU = b8PU + b9A + ε5

ε*n*: *n*=1–5, error term

The direct and indirect connections between the dimensions are represented with numbers indicating the standardized regression coefficients between every two dimensions. All the regression lines are significant with good *R*^2^ values. In addition, the path analysis results also help to find out which one of the five antecedent dimensions has the greatest influence on the ASU of dry-mix mortar. As indicated by the analysis results, PU is the core of the model in this research with a direct effect of 0.557 on ASU. Together with a total indirect effect of 0.275 (0.499 × 0.612), PU has a total effect of 0.832 (0.557 + 0.275). This indicates PU has an indirect influence (0.275) via A on ASU, still less than its direct effect on ASU (0.557). In other words, to promote actual use of dry-mix mortar in the construction industry, enhancing the perceived usefulness of dry-mix mortar is the most efficient method.

## 5. Conclusions

Through the questionnarie sruvey and case study of a typical residential building in Taiwan, it is found in this study that dry-mix mortar is a green material helpful in reducing carbon emissions. Just using dry-mix mortar instead of traditional cemement mortar in the plastering of all the buildings in Taiwan alone can result in a signficantly massive carbon emission reduction. According to our findings, to ensure the effective and efficient promotion of dry-mix mortar, more efforts should be invested first in promoting more awarenes of the good quality and benefits of dry-mix mortar for construction projects among employers and then, after the employers have basic understanding of this material, in promoting awareness of its ease of use.

Even though the quality of dry-mix mortar is much better than that of traditional cement mortar, most companies are cost-consciuous and prefer traditional cement mortar because of its comparatively lower costs. However, dry-mix mortar can help to reduce material losses and the amount of required mortar. For example, the stregnth of a 3 mm-thick structure using dry-mix mortar is equal to that of a 5 mm-thick structure using traditional cement mortar. Therefore, the total costs of dry-mix mortar and its traditional counterpart are very simliar when atually used in construction. It is suggested in this study that, when promoting this new material, more focus can be placed on building standard construction procedures and promoting the right method of using it in construciton work in order to give people a correct idea about the total costs of dry-mix mortar and to encourage them to become more willing to use and promote it. Engery conservation and carbon emssion is an important task for everyone worldwide. It requires the development and a wide application of green materials. It is hoped that, through this research, the promotion of dry-mix mortar can be implemented more efficiently and effectively while the model developed in this study can provide helpful references for future research and promotion of other green materials.

## References

[1] Suga M., Almkvist E., Oda R., Kusaka H., Kanda M. (2009). The impacts of anthropogenic energy and urban canopy model on urban atmosphere. Annu. J. Hydraul. Eng..

[2] Hsueh S.-L. (2012). A fuzzy utility-based multi-criteria model for evaluating households’ energy conservation performance: A Taiwanese case study. Energies.

[3] Su M., Liang C., Chen B., Chen S., Yang Z. (2012). Low-carbon development patterns: Observations of typical Chinese cities. Energies.

[4] Ding G.K.C. (2008). Sustainable construction—The role of environmental assessment tools. J. Environ. Manag..

[5] Quadrelli M., König F., Roos M., Stadtmueller S., Weyershausen B. New powdery water repellents for dry mortar applications. http://www.construction-chemicals.com/product/construction-chemicals/Documents/drymix-conference-yearbook-2007.pdf.

[6] Winter C., Plank J. (2007). The European drymix mortar industry. ZKG Int..

[7] Raymond W. Advanced Dry Mortar Technology for Construction Industry. http://www.psdas.gov.hk/content/doc/2002-1-20/Day%202%20-%20Ir.%20Raymond%20WAN%20-%202002-1-20.pdf.

[8] Michael V.K. (2011). Admixture dosing equipment for modern dry premix mortar plants. Indian Concr. J..

[9] Bayer R., Lutz H. (2003). Dry Mortars. Ullmann’s Encyclopedia of Industrial Chemistry.

[10] Taylor M., Tam C., Gielen D. (2006). Energy Efficiency and CO_2_ Emission Reduction Potentials and Policies in the Cement Industry.

[11] Lai C.M., Wang Y.H. (2011). Energy-saving potential of building envelope designs in residential houses in Taiwan. Energies.

[12] Davis F.D. (1989). Perceived usefulness, perceived ease of use, and user acceptance of information technology. MIS Q..

[13] Igbaria M., Iivari J., Maragahh H. (1995). Why do individuals use computer technology? A Finnish case study. Inf. Manag..

[14] Szajna B. (1996). Empirical evaluation of the revised technology acceptance model. Manag. Sci..

[15] Adams D.A., Nelson R.R., Todd P.A. (1992). Perceived usefulness, ease of use, and usage of information technology: A replication. MIS Q..

[16] Straub D., Limayem D., Karahanna-Evaristo E. (1995). Measuring system usage: implications for theory testing. Manage Sci..

[17] GaBi LCIA Documentation. http://database-documentation.gabi-software.com/america/support/gabi/gabi-lcia-documentation/.

[18] Nunnally J.C. (1978). Psychometric Theory.

[19] Carmines E.G., Zeller R.A. (1997). Reliability and Validity Assessment.

[20] Litwin M.S. (1995). How to Measure Survey Reliability and Validity.

[21] Hair J.F., Anderson R.E., Tatham R.L., Black W.C. (1995). Multivariate Data Analysis with Reading.

[22] Avolio B., Yammarino F.J., Bass B.M. (1991). Identifying common methods variance with data collected from a single source: An unresolved sticky issue. J. Manag..

[23] Huang C.F., Hsueh S.L. (2007). A study on the relationship between intellectual capital and business performance in the engineering consulting industry: A path analysis. J. Civil Eng. Manag..

